# Overseas residency rights of Chinese listed firms’ controllers: Impacts on R&D and public welfare donations

**DOI:** 10.1371/journal.pone.0307596

**Published:** 2024-12-16

**Authors:** Haicheng Zhu, Yule Tian, Ke Yang, Saite Wei, Yufei Yin

**Affiliations:** 1 School of Economics, Zhejiang University of Finance and Economics, Hangzhou, China; 2 School of Economics, Henan Institute of Technology, Xinxiang, China; 3 School of Economics and Management, Huzhou University, Huzhou, China; Universiti Pertahanan Nasional Malaysia, MALAYSIA

## Abstract

By developing an intertemporal decision-making model, this paper investigates the impact of the acquisition of overseas residency by a firm’s beneficial owner on the firm’s intertemporal decision-making. By incorporating a discount rate in the model, the paper finds that obtaining foreign residency alters the temporal distributional characteristics of the utility of the beneficial owner, weakening its preference for intertemporal investment behaviors such as R&D and donations. We then conduct an empirical analysis using the propensity score matching method for Chinese listed firms. The results show that firms with overseas residency have significantly lower R&D investment and an increase in philanthropic donation intensity than other firms. This study provides some practical benefit and relevance to government public policymaking: for developing countries such as China, public policies that take into account incentive compatibility will be more conducive to R&D investments and charitable donations by companies that obtain overseas residency rights, thus enhancing their brand value and social well-being.

## 1 Introduction

Scholars have hotly debated whether the right of residence for senior executives abroad should be restricted [1–3]. Recently, the Chinese government amends relevant laws and regulations to properly regulate residency rights. For example, a new provision has been added to the Law of the People’s Republic of China on Administrative Punishment of Officials (2020). It stipulates that managers of state-owned firms cannot obtain permanent residence qualifications or long-term residence permits without approval. Obviously, this regulation conveys to society the Chinese government’s cautious attitude toward the right to reside abroad. However, it does not impose similar restrictions on other non-public officials, which leads to certain differences in the level of regulatory policy.

People with the right to reside abroad have a high tendency to eventually migrate. For emerging economies like China, the innovation capacity and sustainable development of SMEs and private firms are not only related to the stability and social welfare changes within China, but also to the long-term strategic layout of the country. The emigration tendency of senior executives will have a great impact on the innovation and competitiveness of Chinese SMEs. Therefore, both theoretically and practically, it is a crucial issue to find out the impact of the foreign residence rights of actual controllers on R&D and public welfare donations.

However, existing studies generally focus on taxation and asset transfer [4] and neglect the changes in the domestic investment behavior of firms, especially the intertemporal investment behavior after obtaining the right of abode. Therefore, it is difficult to find a suitable basis for formulating relevant public policies. This paper considers these issues from a different perspective.

First of all, we divide corporate cross-period investment behavior into R&D investment and charitable donation. In other words, we examine the impact of the foreign residency right on R&D investment and charitable donation separately. Through the dual discount model, this paper analyzes the role of residency on corporate intertemporal investment behavior from the perspective of time and psychological costs. This approach is more conducive to a discussion of the mechanism and explains why the acquisition of overseas right of abode affects the income distribution characteristics of the actual controllers of firms, thereby affecting their R&D and donation behavior.

Second, we use financial data of 1833 listed companies in China’s A-share market from 2003 to 2020 to test the impact of residency on executors’ behavior. In the empirical application, the conclusions of the model are tested using the propensity score matching (PSM) method. In this paper, we set up a treatment group and a reference group to classify the net effect of the target relatively accurately. This approach analyzes the theoretical results less biased. Finally, some policy recommendations for developing countries to improve their overseas residency regulatory system are put forward.

This study examines whether and how the foreign residence rights of actual controllers affect R&D and charitable donations. It provides some new insights into the existing literature. First, the paper finds that the acquisition of senior executives’ overseas residency rights has changed the time distribution characteristics of individual income. In the model, it is shown that the actual controller’s time preference for the current income has increased, while the actual controller has become "impatient" with the income that needs to wait for a long time. This psychological waiting cost has led to the gradual increase of the discount rate of the controller, which has significantly reduced the enterprise’s preference for R&D and other intertemporal investments. Second, the analysis results based on the data of Chinese listed companies show that the acquisition of the right of abode outside China under actual control significantly weakens the intensity of R&D investment and the charitable donation of the enterprises, which supports the above conclusion from a practical perspective. Based on the above analysis results, this paper believes that some policies should be designed from the perspective of incentive compatibility, but the necessary regulatory and internal control systems are still necessary. The theoretical significance of this study is that there are relatively few studies on the impact of overseas residency on corporate behavior, and this study introduces this variable into the study of corporate R&D investment and charitable donations, which provides new perspectives and ideas for the research in related fields, and helps us to understand the intrinsic connection between corporate governance and corporate social responsibility more comprehensively. This helps to improve the theoretical system of corporate governance and promote the fulfilment and development of CSR. The practical significance of this study is that it helps enterprises adjust their R&D investment strategy according to the characteristics of the actual controller and achieve optimal allocation and efficient use of resources. It also helps enterprises to recognize the importance of actively fulfilling their social responsibilities and giving back to the society through charitable donations and other means, which not only helps to enhance their social image and reputation, but also enhances their sense of social identity and belonging. The results of this study can provide useful references and lessons for government regulators to strengthen the supervision and guidance of enterprises and promote their compliance and healthy development.

The innovation of this paper lies in the fact that previous studies on the de facto controllers of listed firms have mostly focused on their shareholding structure and business strategies, whereas this study explores the impact of the factual controllers’ overseas residency on firms’ R&D and public welfare donations, which contributes to a more comprehensive understanding of the relationship between the de facto controllers’ personal characteristics and their firms’ decision-making. The limitation of this paper is that due to the difficulties in obtaining data from listed companies, this study may not be able to cover all relevant companies, resulting in the representativeness of the sample being affected to some extent. Future research can improve the representativeness of the sample through a wider range of data sources and stricter screening criteria. Meanwhile, when exploring the impact of overseas residency of beneficial owners on corporate R&D behaviors and public welfare donations, it may not be possible to fully control the impact of other potential variables, such as market environment and policy changes. This may lead to some errors in the research results. Future research can reduce this error through stricter experimental design and finer data processing.

The paper is organized as follows: Section 2 is the literature. Section 3 presents the model and hypothesis. Section 4 points out the data, variables, and methodology. Section 5 shows results and discussion. Section 6 is the conclusions and the corresponding policy implications.

## 2 Literature

### 2.1 Managerial heterogeneity and decision incentives

Referring to the principal-agent theory of Grossman and Hart [5] and Johnson & Clotfelter [6],when ownership and control are separated, the individual utility of senior executives will play a decisive role in the operation of the firm. Subsequently, many scholars started to discuss specific heterogeneity, among which, nationality background and cultural differences attracted the attention of relevant scholars [7, 8]. These studies use a variety of qualitative and quantitative tools to examine the influence of senior executives’ nationality, cultural identity and other factors on the choice of corporate behavior, and further clarify the link between senior executives’ micro utility and corporate behavior. Unfortunately, the above literature does not explore the right of residence and corporate investment behavior in depth, so the reference value of policy recommendations is limited.

### 2.2 Right of abode and investment behavior of firms

The purpose of R&D investment is to obtain intertemporal returns, but Huang et al. (2023) argued that the intertemporal nature of investment exacerbates agency problems in firms [9]. That is, shareholders with a real dominant position may sacrifice enterprise value and then convert it into personal returns [10]. However, the foreign nationality and overseas background of senior executives have different influences on the investment behavior of firms. In the short term, the overseas background of senior executives is conducive to the expansion of investment channels. However, in the long run, R&D investment over a long period will affect the risk assessment of senior executives for intertemporal investment. Especially in the context of global economic instability, stable short-term investment behavior may be the optimal choice [11–13]. Therefore, the time value of money and risk sensitivity of senior executives to earnings will be different before and after obtaining an overseas residency. Adan (2018) empirically tested the negative correlation between the change of nationality of senior executives and investment, which reflects the existence of a disincentive effect [7].

### 2.3 Foreign residence rights and R&D incentive motivation

The R&D behavior in firms is arguably the most important long-term investment a firm can make, but it is also the most vulnerable to agency problems due to its unique characteristics, such as high opacity, uncertainty and risk. Existing studies have discussed whether the acquisition of residency rights changes the R&D decisions of senior executives. These studies focus on the changes in the R&D motivation of senior executives before and after the acquisition of the right to abode abroad. Chen et al. (2018) analyze that the acquisition of overseas residence rights makes senior executives have stronger transnational operation motivation [14]. That reflects it is easy for them to accept new technologies and new development modes. Even if there is a conflict of interest, due to the consistency of interests, senior executives will provide relatively positive support to firms, and overseas residence right will encourage enterprises to invest in R&D [15]. However, Qiu et al. (2021) takes the opposite view. He believes that the acquisition of the right to residence makes executives unwilling to bear higher risks and longer income periods, thus it has a restraining effect on the R&D investment of enterprises [3].

### 2.4 Foreign resident status and public welfare donation

There are many documents on public welfare donation. These articles mainly refer to the researches of Lamia Chourou (2023) [16] and others to understand the micro motives of firms’ public welfare donation. Public welfare donation not only directly increases the firm’s monetary income, but also affects the firm’s image, brand awareness and sales. Meanwhile, it has a similar effect to advertising, which can directly affect the firm’s operational efficiency. In the long run, these activities improve the external environment of the enterprise and lay a solid foundation for the sustainable development of the enterprise. Nyuur et al. (2019) used listed companies as the research sample and found that the motives of enterprises for charitable donations are mainly to increase economic benefits [17]. Executives may use charitable donations for tax avoidance and asset transfer after obtaining overseas residency rights. However, the above studies focus on the economic motivation of giving behavior and ignore the non-monetized private benefits of executives. Xue Xia et al. (2019) mentioned that in addition to monetized economic motives, corporate managers’ donation behavior may also be motivated by the maintenance of their political connections [18]. It has also been mentioned that due to environmental regulations, companies may choose to contribute to society through charitable donations in order to improve their public image and social reputation, thus partially mitigating the negative environmental impacts they may have [19]. They found that the main motivation for executives to make public welfare donations is their individual perception of external public opinion pressure. In short, after the executives obtain foreign residency rights, considering the pressure of public opinion and to maintain intangible assets such as corporate image and brand recognition, executives will use the public welfare donations to "cover up". In modern society with highly developed network information, this kind of motivation is much more important. Therefore, in the follow-up research, we will pay more attention to the non-monetary benefits of donations to obtain more objective research results.

### 2.5 The literature review

The above-mentioned papers have laid the theoretical foundation for research on the overseas residency rights of senior executives. However, the author believes that there are some shortcomings in the discussion of the existing literature. First, the existing research generally takes tax evasion, fraud, etc. as the breakthrough point and focuses on the short-term decision-making behavior of the firm. The problem is that the implementation of the above behavior is only a few people, we cannot draw a general rule and the universal significance. Therefore, it cannot be directly used as an explanatory variable. Therefore, this paper chooses R&D input and charitable donation as proxy variables and takes the discount model as a mathematical tool to make a more complete discussion. Second, the empirical method used in the existing literature is still the traditional linear regression method. It has shortcomings in terms of sample timeliness and method robustness. Therefore, in this paper, propensity score matching (PSM) is chosen for a more complete and comprehensive hypothesis testing.

## 3 Model and hypothesis

From the perspective of organizational behavior, listed firms can be regarded as "social production entrusted by the public". The utility function of the actual controllers (executives) has a very important impact on the business decision-making of the company [5]. From a micro perspective, we can divide the actual controller’s utility function into two parts: monetary income and non-monetary income, where monetary income can be divided into "short-term monetary income" and "long-term monetary income". The related research above mentioned tax avoidance and tax evasion, which focus on "short-term monetary gains". In addition, stock withdrawals and stock dividends can also generate monetary income. However, R&D not only requires a large amount of initial investment, but also the benefits are not currently available, which can only be realized in the future through continuous product sales and profit transformation. Therefore, related scholars believe that R&D is a long-term investment behavior of firms, that is, a current decision based on future expectations [20]. Considering the above three aspects, it can be assumed that the monetary income function obtained by the current controller is M = M(g; t; rd). M is a function of g (stock dividend, payout), t (tax avoidance) and rd (research investment).

Another factor that cannot be ignored is the time cost of monetary gains as well as the psychological cost of cross-time decisions. Any investment income has a time lag effect, and R&D investment is a typical cross-time decision behavior. So we consider introducing a method to measure the time cost (opportunity cost), which is to set a fixed discount rate r_1. However, Dellavigna and Paserman(2001) believe that in addition to the time cost of money, decision-makers should also consider the psychological time cost (waiting cost) caused by the inconsistency of cost and benefit periods [21]. According to the study of Qiu et al. (2021) [3], in the sample of firms whose executives have overseas residency rights, decision-makers tend to invest in the short and medium term and obviously lack "patience" for intertemporal investment such as technological innovation.

Therefore, we set a discontinuous discount factor *δ* = (1+*r*_1_+*r*_2_)^−*i*^ as the coefficient of economic benefits, where r_2_ represents the psychological cost of time. It is an increasing function of the discount period i (time). It means the closer to the current period, the lower the psychological cost, and the closer to the long-term psychological cost, the higher the psychological cost. Therefore, the total monetary income function TM of the actual controller can be set as:

$$TM=\sum_{i=1}^{n}\frac{1}{{\left(1+r_{1}+r_{2}\right)}^{i}}M(g;t;rd)$$

We then turn to examine the utility of public donations to the actual controller. Public welfare donations can be seen as "the private provision of public goods". The motivation of the actual controller to make public welfare donations is more complex than that of R&D. On the one hand, the intangible assets such as corporate reputation and brand image that can be brought by donations, which play a similar role to advertising [16] and can bring financial incentives to firms in the future. Therefore, we assume that M = M(g; t; rd; x), where x represents the number of domestic donations. On the other hand, the non-monetized private benefits of entrepreneurs based on their identities, such as political resources, asset protection, and public opinion support, cannot be measured in economic terms. We assume that their non-monetary function is N = N(S(x)), where S(x) is the sense of social identity that donations bring to entrepreneurs. To be closer to reality, we assume that N and S are concave functions subject to the law of diminishing margins.

In summary, we can deduce the utility function U of the actual controller, which is expressed as:

$$U=TM+N=\sum_{i=1}^{n}\frac{1}{{\left(1+r_{1}+r_{2}\right)}^{i}}M(g;t;rd;x)+N(S(x))$$

Next, we find the first-order partial derivatives for R&D (rd) and donation (x) respectively, and simplify the actual controller’s utility to the elasticity of R&D (*η*_1_) and the elasticity of donation (*η*_2_):

$$\eta_{1}=\frac{\partial U}{\partial rd}*\frac{rd}{U}=\frac{\partial M}{\partial rd}\sum_{i=1}^{n}\frac{1}{{\left(1+r1+r2\right)}^{i}}*\frac{rd}{U}$$

$$\eta_{2}=\frac{\partial U}{\partial x}*\frac{x}{U}=\frac{\partial M}{\partial x}\sum_{i=1}^{n}\frac{1}{{\left(1+r1+r2\right)}^{i}}*\frac{x}{U}+N^{\prime }[S(x)]S\prime (x)*\frac{x}{U}$$

Now we introduce the factor of foreign residency rights. When beneficial owners acquire overseas residency, they may be exposed to a wider international perspective and different life circumstances, which may lead to a shift in their focus. In terms of psychological motivation, they may be more concerned with personal or family international affairs rather than just the daily operations of the enterprise. This psychological change may affect their decision-making on corporate R&D investment, which often requires long-term, sustained financial investment and a high level of attention, which may conflict with the new focus of the beneficial owner. The motives of the actual controllers to acquire foreign residency rights mainly include overseas development [22], asset transfer [23], and tax avoidance [24]. No matter what the motivation is, the acquisition of foreign residency rights will bring subjective and psychological heterogeneity changes to the actual controller, although these changes cannot be directly measured with concrete values. But for the final decision-making behavior, it will manifest itself as an increased preference of the actual controller for the time of the current income and becoming “impatient” for gains that need to be waited for a long time [21]. We assume that foreign residency rights lead to the existence of *r*_2_^^^ which makes *r*_2_^^^>*r*_2_, where *r*_2_^^^ represents the psychological cost of time after obtaining the foreign residency rights, and then the total economic income of the actual controller after the acquisition of the foreign residency rights can be represented as:

TM^=∑i=1n1(1+r1+r2^)iM(g;t;rd;x)

Next, we analyze the impact of the foreign residence right on the non-monetary income of the actual controller. In the previous paragraph, we assumed that the sense of identity brought to the actual controller S(x) by the public welfare donations is a monotonically increasing concave function and obeys the laws of marginal decline. To further consider the effect of foreign residency, we assume that after the actual controller obtains foreign residency, the way to acquire the sense of identity is no longer limited to the domestic territory, but has two options: domestic donation and foreign donation. Therefore, we set x as domestic donation and x1 as overseas donations, and the realistic constraint that donations must meet to better fit the real situation is *x*+*x*1≤*x*^0^. In other words, the total amount that the actual controller is willing and able to donate in the current period does not exceed the fixed value *x*^0^. Furthermore, we set the non-monetary income function of the actual controller as:

$$N=N(S(X))+S(x1)\;and\;x+x1\leq x^{0}$$

Combined with the above analysis, without considering the economic benefits of donating abroad, the utility function of the actual controller after obtaining the foreign residency right is as follows:

U^=∑i=1n1(1+r1+r2^)iM(g;t;rd;x)+NS(x)+S(x1))

And we can find that the elasticity of R&D changes as follows:

η3=∂U^∂rd=∂M∂rd∑i=1n1(1+r1+r2^)i*rdU

and

η3−η1=∂M∂rd*rdU(∑i=1n1(1+r1+r2^)i−∑i=1n1(1+r1+r2)i)≤0

For the actual controller, the inequality of *η*_3_−*η*_1_≤0 means that after obtaining the foreign residence, the rational actual controller will have a lower preference for the investment decision for the intertemporal returns of R&D (the elasticity will be smaller). Under the same constraint, the rational actual controller will prefer a shorter discount period, such as g(dividends) and t(tax avoidance), to balance the current monetary income caused by the increase in the psychological cost of time, which is represented by (*r*_2_^^^−*r*_2_). Suppose we further propose a stronger hypothesis: If the funds that can be invested in the short term are fixed in value and duration (expected to turn into immigration in the future), then R&D investments that require a long period and have a higher psychological time cost will be further squeezed and reduced in the current decision. The first hypothesis to be tested in this paper is as follows:

**H1:**The acquisition of foreign residence rights by the actual controller will weaken the R&D investment of the firm.

From the perspective of resource allocation, the actual controller, after obtaining the right of abode in a foreign country or the right of abode outside the country, may devote more resources and energy to activities related to internationalization, such as overseas investment and market expansion. These activities usually require substantial financial and resource support, and the resources of an enterprise are limited. This adjustment of resource allocation may affect the firm’s public welfare donation decision. Public welfare donations are part of CSR, but with limited resources, firms need to make trade-offs between different socially responsible activities. If actual controllers devote more resources and energy to internationalization activities, they may invest less in social responsibility activities such as public benefit donations. In addition, the personal preferences and concerns of actual controllers may also affect firms’ public benefit giving decisions. If they pay more attention to internationalization activities, they may believe that these activities are more reflective of corporate social responsibility, and thus pay less attention to and invest in public welfare donations. For donations, it is also a concave function that obeys the law of diminishing margins. Once the donation has reached a certain value, increasing the identity brought by the donation will not produce a higher marginal utility. In general, if the actual controller has already made a certain amount of domestic donations before obtaining foreign residence rights, the initial value of x will be greater than that of x1, and then the further increase in x will bring far less utility than the increase in x1. So, we can deduce that:

η4=∂U^∂x*xU=∂M∂x*xU∑i=1n1(1+r1+r2^)i+N′[S(x)+S(x1)]S′(x)*xU

According to the properties of concave functions, the inequality must be:

$$\eta_{4}-\eta_{2}\leq 0$$

Moreover, if the actual controllers are eventually transformed into migrants, their national identity satisfaction will be unnecessary, expressed by the formula (N(S(x)) = 0). Even if they cover up for public opinion reasons, their social donations will be reduced in the long run [25]. Therefore, the second hypothesis to be tested in this paper is as follows:

**H2**: The acquisition of foreign residency rights by the actual controller will weaken the firms’ public welfare donation.

## 4 Methodology

### 4.1 Data

Relevant research has focused on the impact of changes in executive nationality and wealth transfer on corporate innovation and competitiveness [3, 4, 7, 14]. Most of them have chosen China as the research sample and emphasize the special nature of executive residency abroad for China’s economic development. Why has research on overseas residency become a social issue with strong Chinese characteristics? The reason is that, as the world’s largest developing country, small and medium-sized enterprises have always been an important pillar of China’s economic growth (approximately 42 million households, accounting for over 99%). The improvement of innovation capabilities of small and medium-sized enterprises plays an important role in the development of emerging economies. However, due to factors such as the system and environment of the home country, when wealth accumulates to a certain extent, entrepreneurs tend to develop overseas to obtain higher economic returns. The improvement of innovation capabilities of small and medium-sized enterprises plays an important role in the development of emerging economies. However, due to factors such as the system and environment of the home country, when wealth accumulates to a certain extent, entrepreneurs tend to develop overseas to obtain higher economic returns.

Generally, the actual controller of SOE is the government asset management department, so the observations are meaningless for the research of this paper. Therefore, we conducted a targeted screening and mainly retained pure private enterprises and private enterprises transformed from SOE, accumulated financial data of 1833 listed companies from 2003 to 2020, with the observations obtained from the financial reports of the companies. Some information on a controlling person’s residency rights status is manually matched and screened through annual reports, and some are also obtained through the official websites of various companies.

### 4.2 Variables

#### 4.2.1 Outcome variables

The R&D investment (rd) set in this article is the proxy variable for the company’s R&D investment in the current year. This includes various expenses incurred by enterprises in the process of researching new products, technologies, materials, and processes, as well as labor remuneration such as salaries and benefits for related research and development personnel. Therefore, in the current accounting standards in China, R&D investment, as an important expense, is usually separately listed in the company’s financial statements and belongs to accounting items such as "R&D investment", "R&D expenditure", and "development expenditure". The data source used in this article is the publicly available financial annual reports of each listed company, based on the amount of expenses or costs recognized in the current year, which is the core explanatory variable (logarithmic) of this article. The public welfare donation in this paper is directly obtained from the financial annual reports published by various listed companies. It should be noted that, based on the theoretical analysis process and examination of real cases, we believe that foreign residency rights have an inter-temporal influence on the public welfare donation decision-making of enterprises. To examine the “marginal” nature of long-term changes, and to compare changes before and after obtaining foreign residency rights, we focus on the donation increment (donate).

#### 4.2.2 Treatment variable

The treatment variable in this paper is foreign residency rights (abord). According to the definition of Zhang et al. (2016) [23], foreign residency rights can be understood as the right of non-citizens to temporarily or permanently reside in a certain country, which is granted by third countries or regions. For example, a "green card" issued by a certain country is typical proof of residency. China does not allow residents to have dual or multiple citizenships, so citizens who have the equivalent of a foreign residence permit have the right to facilitate entry. It is a certificate that must be obtained for overseas immigration. Domestic and foreign scholars have discussed the motivation of the actual controller to obtain foreign residence right. It can be summarized as asset transfer, tax avoidance, environment and education, and so on. In general, it is based on the subjective motivations of individuals. The actual controller’s foreign residency right is not caused by internal factors of the firm, which is relatively strict exogeneity. The main source of information comes from investor relations announcements and annual reports on the relevant website (Tidal News) published by companies.

#### 4.2.3 Control variable

The relationship between R&D, donation, and foreign residency right is relatively complex. To avoid endogeneity issues caused by omitted variable bias, the control variables used in this article mainly consider the following aspects. Firstly, all control variables can reflect the individual characteristics of the company, and there is no contradiction or repetition between variables. Secondly, all control variables should correlate with the explained variable R&D investment and donation, And the acquisition of overseas residency rights by executives will be associated with control variables.

After screening, the applicable control variables in the R&D model are shown in **Table 1**. Among them, variables such as minority shareholders’ equity (minints) and earnings per share (eps) measure earnings belonging to shareholders, which directly affect the company’s research and development and other intertemporal investment decisions. The remaining variables such as quick ratio (quration), the net return on assets (roe), total assets (talast), total liabilities (talbet), main business income (mainbus), sales income (saleincm), and the total revenue (toperate) are used to control variables such as the company’s size and operational level that may affect the company’s decision-making. Due to the large individual bias of the enterprise, to avoid strong data fluctuations caused by heteroscedasticity, this article conducted logarithmic processing on the above variables (since earnings per share itself fluctuates less, logarithmic processing was not performed).

**Table 1:** Variable definitions in R&D.

Variables	Variable name	Abbreviate
Dependent variable	R&D	rd
Treatment variables	Foreign residency right	abord
Control variables	Capital reserve	capitalr
	Minority shareholders’ equity	minints
	Earnings per share	eps
	Quick ratio	quota
	Net return on assets	roe
	Total assets	talas
	Total liabilities	talbet
	Main business income	mainbus
	Sales income	saleincm
	The total revenue	toperate

Although R&D and donations have similar income characteristics, their decision logic and economic motives are very different. Therefore, the result will lose its unbiasedness if we use the same group of control variables. In the donation model, some specific control variables groups are introduced before the matching. Among them, due to the significant differences in companies between different sectors, we introduce the ChiNext (chinext) as a covariate. The total share capital (gencaptial) and circulating share capital (capcircu) are further supplementary controls on the differences in the size of a company’s share capital. The above three variables play a crucial role in the correctness of the matching results. The impact of controlling residency rights on company decision-making is directly related to their discourse power in the company. Therefore, we choose the proportion of first shareholders (first), number of board members (number), and independent directors (independent) to reflect the decision-making power of actual controllers in different companies. In addition, we have also introduced total assets (totalasset), total liabilities (totaldebt), number of employees (emploee), and fixed assets (fixedasset), which help distinguish enterprises of different sizes and effectively avoid confusion and matching between large and small enterprises. Due to the differences in charitable donations among enterprises with different operating efficiencies, we also selected common business variables such as sales tax and surcharges (saletax), income tax rate (incmtrate), and so on. In the dimension of political relevance, we need to consider the impact of the founder’s political identity (poffoun) on the company’s donation behavior. The supplementary control of multiple dimensions mentioned above will be beneficial for the accuracy of matching results. Compared to traditional linear estimation, it can better strip out the interfering factors, thus obtaining the net effect of overseas residency on donations. The specific algorithms are summarized in **Table 2**.

**Table 2:** Variable definitions in donation.

Variables	Variable name	Abbreviate
Dependent variable	Public welfare donation	donate
Treatment variable	Foreign residency right	abord
Control variable	ChiNext	chinext
	Total share capital	gencaptial
	Circulating share capital	capcircu
	Shareholding ratio of the first shareholder	first
	Number of directors	number
	Number of independent directors	independent
	Number of employees	emploee
	Total assets	totalasset
	Total liabilities	totaldebt
	Fixed assets	fixedasset
	Business tax and surcharges	saletax
	Income tax rate	incmtrate
	Inventory turnover rate	rateturnover
	Accounts receivable turnover rate	recvratio
	Total Asset turnover	totalasturn
	Tax refund	taxreturn
	Founder’s political identity	poffoun
	Main revenue	mincome

### 4.3 Model settings

To identify the crowding out effect of research and development investment and donation on executives with overseas residency rights, combined with micro mechanism analysis, the benchmark econometric model of this article is set as follows:

$${rd}_{it}=\alpha_{0}+\alpha_{1}{abroad}_{it}+\beta X_{it}+\gamma_{t}{+\mu }_{i}+\varepsilon_{it}$$ (1)

$${donate}_{it}=\alpha_{2}+\alpha_{3}{abroad}_{it}+\beta X_{it}+\gamma_{t}{+\mu }_{i}+\varepsilon_{it}$$ (2)

In the above equation, rd_it_ and donate_it_ are the R&D investment logarithmic form and donation increment of the sample enterprise in year t. The abroad_it_ is whether the controlling shareholder, chairman, and other senior executives of the enterprise in year t have obtained the right of abode, and are assigned a value of 1 after obtaining and obtaining it. Otherwise, it is 0. X_it_ is a series of control variables, aimed at alleviating endogeneity problems caused by measurement errors, sample principles, etc. *γ* t is a time fixed effect, *μ* i represents an individual fixed effect that does not change over time, and *ε* it is a random disturbance term. In the following text, when testing other effects of policies, appropriate adjustments will be made to the model.

### 4.4 Descriptive statistics

**Table 3** shows the summary statistics of key variables. Our sample consists of firm-year observations from 2003 to 2020. The mean value of abord is 0.128 with a standard deviation of 0.334 indicating that about 12.74% of observations in our sample have foreign residency rights. The maximum value of rd is 9.636, the minimum value is 0, the maximum value of donate is 10904.08, and the minimum value is -0.700, indicating a significant gap in R&D and public welfare donation among different firms. Therefore, it is necessary to explain the reasons why there is such a huge difference between the firms’ R&D and public welfare donation.

**Table 3:** Summary statistics of key variables.

Variables	N	Mean	Std	Minimum	Maximum
rd	15,323	8.637	0.998	0	9.636
donate	11,896	78.079	332.666	-0.700	10904.080
abroad	18,025	0.128	0.334	0	1
capitalr	21,011	8.936	1.013	0	9.948
minints	21,011	8.355	1.188	0	9.736
eps	21,011	0.252	0.692	-21.860	10.950
quratio	20,858	0.281	1.016	-9.210	5.191
roe	17,938	-2.670	0.995	-9.210	5.075
talast	21,011	8.909	1.160	0	9.948
talbet	21,011	8.913	1.133	0	9.948
mainbus	21,011	8.864	1.264	0	9.943
saleincm	21,011	8.893	1.172	0	9.946
totoperat	21,011	8.893	1.172	0	9.946
nofindir	18,025	0.389	1.028	0	8
longtmin	21,011	6.912	2.455	0	9.501
chinext	15,536	-	-	0	1
gencaptial	15,542	5.491	9.285	0.350	364.854
capcircu	15,542	3.856	7.418	0	295.518
first	11,896	32.970	14.371	0.820	89.990
number	11,896	2473.099	5937.746	0	196026
emploee	15,467	8.430	1.638	0	18
independent	15,470	3.080	0.546	0	6
totalasset	15,515	66.899	1543.302	0.001	167836
totaldebt	15,516	34.977	703.080	-0.020	55438
fixedasset	11,876	6.433	14.959	0.000	333.750
saletax	15,396	0.351	1.676	-0.292	90.050
incmtrate	11,795	19.544	6.854	0	33.000
rateturnover	11,688	58.230	1961.861	0	120530
recvratio	11,760	257.582	9310.887	0	851306.200
totalasturn	11,878	0.659	0.569	-0.092	12.373
taxreturn	567	287.138	817.592	-262.610	13934.540
poffoun	14,642	0.571	0.495	0	1
mincome	11,883	20.831	57.287	-3.872	1423.110

### 4.5 Methodology

The method of propensity score matching was first used to deal with data from non-random biological experiments, and then widely used in the social sciences. Specifically, the propensity score matching method (PSM) is a method of control group in the sample by matching the treatment group, so as to reduce the unavoidable confounding variables and data bias in the observations. The specific implementation steps of the propensity score matching method are as follows: the multidimensional measurable variables of individuals in the sample were compressed into one-dimensional, and then the most similar individuals in the sample were found through functions (nearest neighbor, radius, etc.). Finally, we processed and estimated the average treatment effect. We set the years in which the actual controllers obtained the right of foreign residency (abord = 1) as the treatment group and the years in which the actual controllers did not obtain the right of foreign residency (abord = 0) as the reference group. We then matched the data from the two groups using the logit regression method.

## 5 Results discussions

### 5.1 The basic regression

The basic regression in the article uses the OLS estimation method to estimate the model (1). In **Table 4**, columns (1) and (2) measure the relationship between executives’ overseas residency rights and R&D investment. The results indicate that there is a negative correlation between executives’ overseas residency rights and R&D investment. Columns (3) and (4) measure the relationship between executives’ overseas residency rights and public welfare donations. It shows that there is also a negative correlation between them.

**Table 4:** The basic regression.

Variables	R&D		Donate	
	(1)	(2)	(3)	(4)
abroad	-0.049*	-0.049*	-24.607***	-30.972
	(0.026)	(0.027)	(7.274)	(38.371)
cons	8.466***	10.352***	89.743***	-301.006**
	(0.196)	(0.256)	(4.122)	(171.562)
control	NO	YES	NO	YES
Year	YES	YES	YES	YES
Industry	YES	YES	YES	YES
N	14,257	12,699	9315	464
R^2^	0.004	0.023	0.005	0.1991

Notes: the standard error in parentheses

* p < 0.1

** p < 0.05

*** p < 0.01.

### 5.2 Foreign residency rights and R&D

To test the robustness of the basic regression results, this paper uses the propensity score matching (PSM) method in the counterfactual analysis framework to test robustness. In this article, the economic and political environment of domestic listed companies is completely the same, and the sample size is relatively large. Therefore, there is sufficient capacity for near-class matching to eliminate deviations caused by industry factors and individual enterprise factors. Enterprises, where executives have obtained residency rights (abroad = 1), are treated as a reference group. The years before the controller has obtained residency rights and the year before the controller has obtained residency rights are set as a reference group. The two sets of data are matched using the logit regression method, and the control variable group used in the matching process is consistent with the basic regression. After matching, most of the observed values are within the common range, and the matching results balance the sample data well. In addition to the nearest neighbor matching (N), this paper also uses radius calipers (Cal), kernel density matching (Kernel), and spline matching (Spline) to jointly test the robustness of the results. The common values of various methods are consistent with the standardization deviation. The ATT, ATE, and corresponding standard errors are summarized in **Table 5**.

**Table 5:** R&D grouping treatment effect.

Matching method	rd	
	ATT(diff)	ATE(diff)
Nearest neighbor matching (1)	-0.095***	-0.066
	(0.036)	
Nearest neighbor matching (4)	-0.060**	-0.058
	(0.030)	
Radius matching (caliper:0.01)	-0.049*	-0.048
	(0.027)	
Kernel matching	-0.051*	-0.050
	(0.027)	
Spline matching	-0.048*	-0.045
	(0.028)	

Notes: the standard error in parentheses

* p < 0.1

** p < 0.05

*** p < 0.01.

We mainly focus on the ATT treatment results of the R&D group. As we can see from **Table 5**, ATT are stable under the matching methods and the corresponding parameters, the significance level is maintained within 10%, and the standard error is within 0.1, which can significantly reject the hypothesis that there is no difference between the treatment group and the control group. The result is a stable and reliable net effect that excludes other factors. In an economic sense, it can be interpreted that the R&D for enterprises whose actual controller has foreign residency rights (treatment group) is, on average, 4.8%-9.6% lower than that of enterprises whose actual controller does not have foreign residency rights (control group). In Table 1, the result of ATE is stable, which had a certain numerical difference with ATT, but the positive and negative directions of these are the same. Therefore, according to the above empirical results, the hypothesis H1 is confirmed.

It takes a long time for the benefits of R&D to be translated into cash profits. In other words, for the actual controller, there is a time inconsistency between input and output. It takes a long time for the benefits of R&D to be translated into cash profits. In other words, for the actual controller, there is a time inconsistency between input and output. Therefore, when the actual controller of the firm obtains the right of residence abroad, the actual controller will become "impatient" for future income in China due to the motivation of overseas investment or asset transfer. Dellavigna and Paserman(2001) state that such "impatience" is a typical psychological time cost, which will have an important impact on the current decision-making of investors [21]. On the other hand, since the actual controller obtains the foreign residency right, the actual controller prefers the investment that can bring the current monetary income [26]. Driven by that motive, many actual controllers or executives tend to avoid taxes and manipulate shares into cash. The recent high-profile cases of overseas asset transfer by shareholders of listed firms are the strongest evidence of the above conclusion.

### 5.3 Foreign residency rights and public welfare donation

For rational individuals, the original motive for public welfare donation is to maximize their utility. To simplify the analysis, we divide the utility provided by donation into monetary and non-monetary benefits, both of which are not easy to observe directly. When the actual controllers perceive the social pressure, they will use the donation behavior to "cover up", so the traditional linear regression cannot identify the net effect of the foreign residence right on public welfare donation. We need to use the PSM method for counterfactual estimation and the method of setting a reference group for comparison to eliminate other confounding factors.

Similarly, we use the nearest neighbor and radius caliper matching methods for processing, and the results are shown in **Table 6**. Under the nearest neighbor matching, the value of ATT in the public welfare donation group is stable between -20 and -38, the value of ATT is stable at -30. Combined with the practical meaning of the data, it can be interpreted that the increase in public welfare donations for enterprises with overseas residency rights by the actual controller is about 200000 to 380000 yuan lower than that of enterprises without residency rights. Four different matching results indicate that the conclusion is stable and reliable. Therefore, hypothesis H2 that the actual controller has overseas residency rights will weaken the domestic public welfare donations by the enterprise is also valid. However, unfortunately, the highest significance level of ATT in this group is only 15%, so this conclusion is not as strong as hypothesis H1.

**Table 6:** Public welfare donation grouping treatment effect.

Matching method	Public welfare donation		
	ATT	ATE	SE
nearest neighbor matching (1)	-37.138	-32.300	25.385
	(-1.46)		
nearest neighbor matching (2)	-24.150	-31.665	26.518
	(-0.91)		
radius matching(caliper:0.01)	-29.036	-34.468	26.358
	(-1.10)		
radius matching(caliper:0.05)	-21.607	-27.577	23.670
	(-0.91)		

Notes: t statistics in parentheses, * p < 0.1, ** p < 0.05, *** p < 0.01.

From the perspective of the actual controller, the economic benefits of donations are the same as those of R&D investment, which belong to future benefits and are affected by the psychological cost of time. If the actual controller gives up the expectation of long-term development at home or intends to emigrate completely, then the public welfare donation at home becomes "unnecessary". Although it plays a “covered-up” role in the face of public pressure, the marginal quantity will still decrease in the long term. On the other hand, the sense of identity gains from the public welfare donation occurs in the current period and the marginal utility decreases. If entrepreneurs with foreign residency right continue to increase their domestic donations, they can no longer gain a higher sense of identity, nor can they gain higher non-monetary benefits. On the contrary, if donations abroad are easy to win public support in the foreign country, it will be easy to obtain non-monetary benefits such as political resources and asset protection. Therefore, in reality, we can see that many entrepreneurs still insist on overseas donations even under the pressure of domestic public opinion. The reason is that overseas donations have gained a higher sense of identity and honor in the current period. Although its marginal utility will decrease with the increase of overseas donations, it still can meet the donor’s requirement of maximizing utility in the current period.

### 5.4 Robustness tests

The above empirical results demonstrate the inhibiting effect of the actual controllers’ foreign residency rights on firms’ R&D and donations. To further ensure the robustness of the results, in this paper we use the method of replacing the data samples and control variables. The specific procedure is as follows: (1) Delete the missing cross section without foreign residency. Due to the late information on the disclosure year of the foreign residency right and the large number of enterprises restructured from state-owned enterprises, there is a large amount of missing data on the foreign residency right. To maintain the integrity of the data, we reserved all the foreign residency rights data, but in the following robustness test, we will have a cross-sectional screening for all the missing data. (2) Replacing some covariates. Theoretically, R&D and donation decisions are affected by the control variables mentioned above. However, in the real market environment, the environment of firms’ R&D and donation decisions is complex and constrained by multiple factors in reality. Therefore, we appropriately handle and replace some financial statistical calibers to investigate and examine the robustness of the empirical results when other factors are controlled. Due to space limitations, we report only the most critical ATT results, as shown in **Table 7**.

**Table 7:** Robustness test summary list.

Matching method	R&D			Public welfare donations		
	ATT	ATE	SE	ATT	ATE	SE
nearest neighbor matching(1)	-.010***	-.004	0.004	-.001*	-.0001	0.0003
	(-2.84)			(-1.76)		
nearest neighbor matching(2)	-.005**	-.003	0.003	-.0004*	-.00006	0.0003
	(-2.09)			(-1.59)		
radius matching(caliper:0.01)	-.004**	-.003	0.018	-.0003	-.00008	.0002
	(-2.04)			(-1.37)		
radius matching(caliper:0.05)	-.002	-.002	0.016	-.0002	-.00002	0.0002
	(-1.37)			(-0.81)		

Notes: t statistics in parentheses

* p < 0.1

** p < 0.05

*** p < 0.01.

As shown in **Table 7**, after replacing the data and some of the covariates, the ATT value of the R&D group changed, but the overall range of variation was not large. The significance level and correlation were the same as the results in **Table 4**, which passed the robustness test and supported hypothesis 1. Treating the donation group in the same way, the significance level became smaller, but the estimated value of the overall ATT is negative and consistent with the results in **Table 7**. In general, the results are robust. That means it also passed the robustness test and confirmed hypothesis 2.

## 6 Conclusion and policy implications

This paper examines whether firms whose ultimate controllers have foreign residency rights are more likely to change their decisions on R&D investment and public welfare donations. We find a negative association between foreign residency and the intensity of R&D investment and public welfare donations. The acquisition of the foreign residency right by the actual controller will weaken the intensity of corporate R&D investment and corporate public welfare donations. It is because the utility function of the actual controller (agent) will change with the acquisition of the foreign residency right. As the actual controller obtains the foreign residency right, the preference for future benefits from R&D and donations will be weakened, and the marginal utility brought by foreign donations is higher than domestic donations. Therefore, in the short term and when resources are controllable, the actual controller of the corporation will inevitably weaken and squeeze domestic R&D activities and donations. We put forward the perspective of incentive compatibility as follows:

Firstly, redesign laws and regulations on the foreign residency right based on "incentive compatibility". As the actual controller of the listed firm like the agent of public wealth, blindly prohibiting and restricting the foreign residency right may damage the production enthusiasm of groups with a high marginal contribution. Therefore, we suggest designing relevant laws and regulations from the perspective of incentive compatibility. For example, we should expand the tax exemption and incentive policies for entrepreneurs to transfer their overseas income and assets to the domestic market, and give them priority in currency exchange and administrative examination and approval. It is necessary to provide policy support in the examination and approval of tax credits, deductions, scope, and so on for firms that use overseas funds to make domestic public welfare donations. In addition, it is important to consider including public donations in the credit investigation system to enhance the donor’s sense of social identity. As mentioned above, non-monetary income is an important motivation for donation. However, in reality, we find that the public opinion environment in China does not pay continuous attention to corporate donations, which reduces the actual controllers’ satisfaction with social identity and other psychological satisfaction gains. Therefore, we suggested that the "ID card" system for public welfare donations should be established, and the donation behavior should be included in the scope of business credit investigation and personal credit investigation. Then, establish a higher degree of donation system of integration with social life, which can also enhance the donors’ sense of honor and social identity.

Secondly, establish the "risk warning system" and "trigger strategy" of the foreign residency right. In general, for the public, the information on the change of decision-making preference after the actual controller obtains the foreign residency right is incomplete. According to the conclusion of this paper, we believe that it is necessary to further track the risks of enterprises whose actual controllers have obtained the foreign residency right to break the asymmetric information. Considering the current constraints of the legal environment of the securities market, we believe that the system can be designed with the following ideas: adding the tracking mechanism of enterprise operation decision-making. For example, if the firm continues to reduce its R&D investment and public welfare donations for more than three years, the enforcement agencies should require the firm to provide reasonable explanations and require to disclose its cross-border investment. If it cannot provide reasonable explanations, enforcement agencies should provide risk warnings to social investors promptly. In addition, once the company has cut R&D investment and public welfare donations beyond the specific value, an economic investigation is required. And if the actual controller is found to have violated regulations on international capital transfer and tax evasion, it should be punished by law. Meanwhile, the criminal costs should be greater than the income that can be obtained by transferring assets. The purpose of these two methods is to break the information asymmetry and create a cost deterrent to crime in the system.

Thirdly, in order to ensure transparency and compliance in the operation of the organization, it is particularly important to establish a sound system of internal supervision and control. Although external supervisory measures play an indispensable role in preventing and correcting irregularities, external supervision in response to the issue of offshore residence is often confined to ex post facto treatment, i.e., supervision and penalties are imposed after the fact of impairment of the relevant welfare has occurred. Although this kind of ex post facto regulation can have a certain warning and punitive effect, it cannot prevent and avoid the occurrence of problems at the source, and it also has problems such as high cost and low efficiency. Therefore, compared with this kind of ex post facto supervision, strengthening and improving internal control is undoubtedly a more effective policy choice. An internal control system can minimize the risk of non-compliance and loss by ensuring that decisions and actions are in line with the interests and objectives of the organization through rigorous process and system design before problems occur. To address the issue of offshore residency, this paper suggests deep optimization of the existing internal control system. Firstly, the scope of authority of independent directors should be increased to enable them to participate more fully in the major decisions and daily management of the company, and to give full play to their professional advantages in supervision, consultation and decision-making. At the same time, a sound accountability system for independent directors should be established to clarify their duties and rights and ensure that they are able to assume corresponding responsibilities while exercising their powers. In addition, in order to further improve the relevance and effectiveness of internal control, it is recommended that a third-party assessment system for overseas investment risks be established. This system can employ a professional third-party organization to conduct comprehensive risk assessment and monitoring of the company’s overseas investment activities, timely identify and warn potential risk points, and provide scientific basis for the company’s decision-making. Through the design of such a system, the company’s internal control capability can be further strengthened and its ability to respond to and manage the issue of overseas residence can be improved.

## Supporting information

S1 Data The data serves as the basis for the empirical analysis in this article. (RAR)
